# Organoid: Next-Generation Modeling of Cancer Research and Drug Development

**DOI:** 10.3389/fonc.2021.826613

**Published:** 2022-01-28

**Authors:** Jungang Liu, Xiaoliang Huang, Lihaoyun Huang, Jinlian Huang, Dingyu Liang, Lixian Liao, Yuqing Deng, Lihua Zhang, Beibei Zhang, Weizhong Tang

**Affiliations:** ^1^ Division of Colorectal & Anal Surgery, Department of Gastrointestinal Surgery, Guangxi Medical University Cancer Hospital, Nanning, China; ^2^ Institute of Biomedical Research, Yunnan University, Kunming, China

**Keywords:** colorectal cancer, organoid, construction methods, drug screening, personalized treatments

## Abstract

Colorectal carcinoma is a highly prevalent and heterogeneous gastrointestinal malignancy. The emergence of organoid technology has provided a new direction for colorectal cancer research. As a novel-type model, organoid has significant advantages compared with conventional tumor research models, characterized with the high success rate of construction and the high matching with the original tumor. These characteristics provide new possibilities to study the mechanism of colorectal carcinogenesis and improve the treatment effects. The present literature would mainly summarize the characteristics of tumor organoids and the up-to-date technique development of patient-derived organoids (PDOs) and application in colorectal cancer.

## Background

Colorectal cancer (CRC) is a highly prevalent and incurable tumor in the population, which is recognized as the third riskiest cancer of death worldwide and the second most deadly cancer in the United States (1). There are some interactions among the causes for CRC development, involving genetic features, microbial effects, and cell growth and metabolism. The majority of colorectal carcinogenesis not induced by familial inheritance is often attributed to genetic and epigenetic mutations of intestinal epithelial cells and aberrant activations of specific signaling pathways that mediate self-renewal and metabolism of intestinal stem cells (2, 3). For example, in the Wnt/β-catenin pathway, increased secretion of Wnt ligand proteins increases the interaction with cell surface receptors, inducing a failure of the intracellular degradation of β-catenin by the complex composed of APC, Axin, GSK-3-, and other proteins. Subsequently, β-catenin accumulates intracellularly and migrates into the nucleus, promoting the transcriptional expression of downstream target genes involved in CRC development (4). In addition, abnormal activation of EGFR/RAS/RAF/ERK, PI3K/AKT, and TGF-β pathways can also be a possibility for colorectal carcinogenesis (5).

Regarding traditional models in CRC research, APC(Min/+) mice are mainly used to study the effect of the interaction between APC and other related genes and pathways on the origin and progression of intestinal adenomas (6, 7). Adenomas cultured in APC(Min/+) mice could maintain their tissue and cell atypia, but no further infiltration could be found in pathological sections. (8), causing that the role of APC (Min/+) mice was described to mimic precancerous and early-stage CRC as well as to study the prevention of CRC initiation, which should be supplemented by other models for the prognosis of advanced CRC.

In addition, cell lines, patient-derived xenograft (PDX), and tumor organoids were created for studies of each stage of CRC. However, the limitation of cell lines and PDX lies in their loss of heterogeneity of CRC genes, which was inaccurate in the study of advanced CRC (9, 10). Due to the necessity of improving the incidence and prognosis of CRC research, models that better represent the cancer microenvironment are constantly being created. Concerning the tumor organoid, it is a 3D structural model, combined with 3D culture technology and constructed by inducing tissue stem cells to differentiate into functional cell clusters *in vitro*. It has apparent advantages, such as simple operation, fast proliferation, short culture cycle, high success rate, high reducibility to the physiological properties of tumor tissues, and self-renewal ability.

Organoids can be used as a new reference tool for tumor research to a certain extent. Since the team of Sato successfully cultured Lgr5 intestinal stem cells into crypt-villus structures of the small intestine *in vitro* in 2009 (11) and patient-derived organoids (PDOs) in colon cancer in 2011 (12), many research teams have successively studied epithelial enriched tumor organoids such as liver (13), pancreas (14), esophagus (15), and stomach (16) and found that these models match well with the original tumor. The high match of these organoid models was found to successfully reflect the heterogeneity of the tumors after proliferation and differentiation. As the first successfully cultured PDO, CRC organoids have vast application prospects in establishing a tumor organoid biospecimen bank, studying the mechanism of CRC development, screening antitumor drugs, predicting drug response, and making personalized treatments. This paper mainly summarizes the construction and application of colorectal cancer organoids.

## Characteristics of Organoids

**Figure 1:**
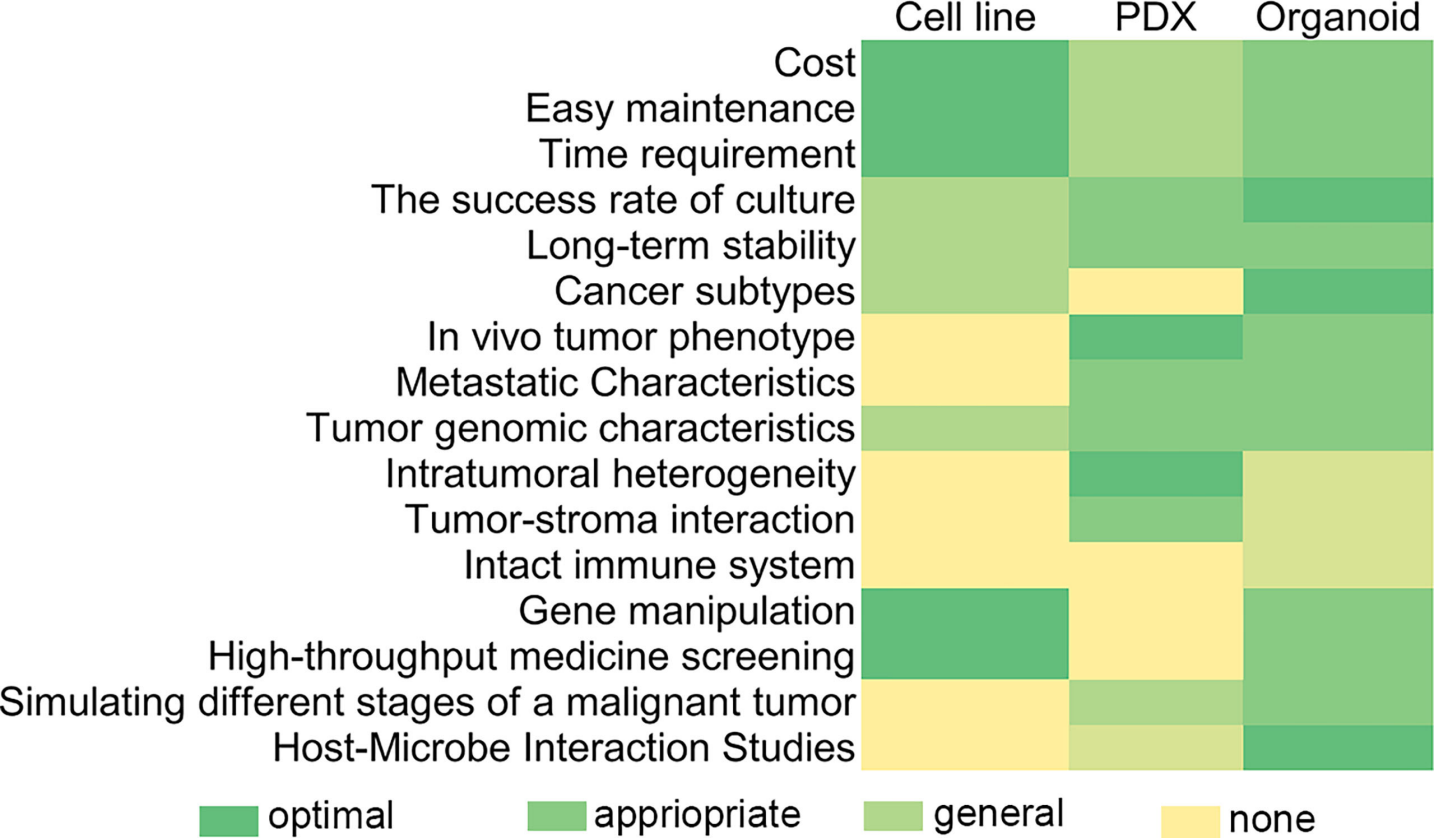
Comparison and evaluation of organoid with traditional tumor research models in the feasibility of cultivation conditions and range of clinical and genetic research.

In comparison with traditional cancer research models like cancer cell lines and PDX models, tumor organoids have multiple advantages in terms of basic criteria for preclinical models, representation of primary tumors, and application in clinical research (**Figure 1**).

Cancer cell lines are simple in culture conditions and easy to maintain, have broad utility and lower reagent costs (17), and can proliferate indefinitely *in vitro* but have more external mutations during culture, such as acquisition and loss of genetic information and altered growth and invasiveness (18, 19). Compared to cell lines, establishing tumor organoids is much cost-effective but has a faster proliferation rate, shorter culture cycle, good maneuverability, high ratio of culture success, and stable transmission.

In terms of representation of the primitive tumor tissues, PDX models retain the main features of the original tumor but lack a complete immune system; cell lines usually do not cover the full genetic spectrum of tumor types (20) and fail to reflect tissue/tumor structure, cell-type composition, etc. In contrast, PDOs show a high degree of concordance with tumors from matched patients in terms of morphological and mutational features and a similar distribution to the mutational profile contained in the cancer genome map, along with similar profiles of DNA copy number compared to parental tumors (21–23). Because of the better recapitulation of original tissue traits in an organoid, it can mimic clinically relevant drug responses that cell lines and PDX models cannot (17).

Organoids can also reproduce cancer development and show substantial differences in molecular characteristics and preclinical responses of different tumors to different treatments (21). Moreover, they can support the survival and growth of anaerobic and aerobic bacteria (24); therefore, they can be used to study the pathogenesis of infectious diseases and to analyze various aspects of the “chain of infection” model in (pre)clinical studies (25).

## Development of CRC Organoids

### Materials and Methods

**Figure 2:**
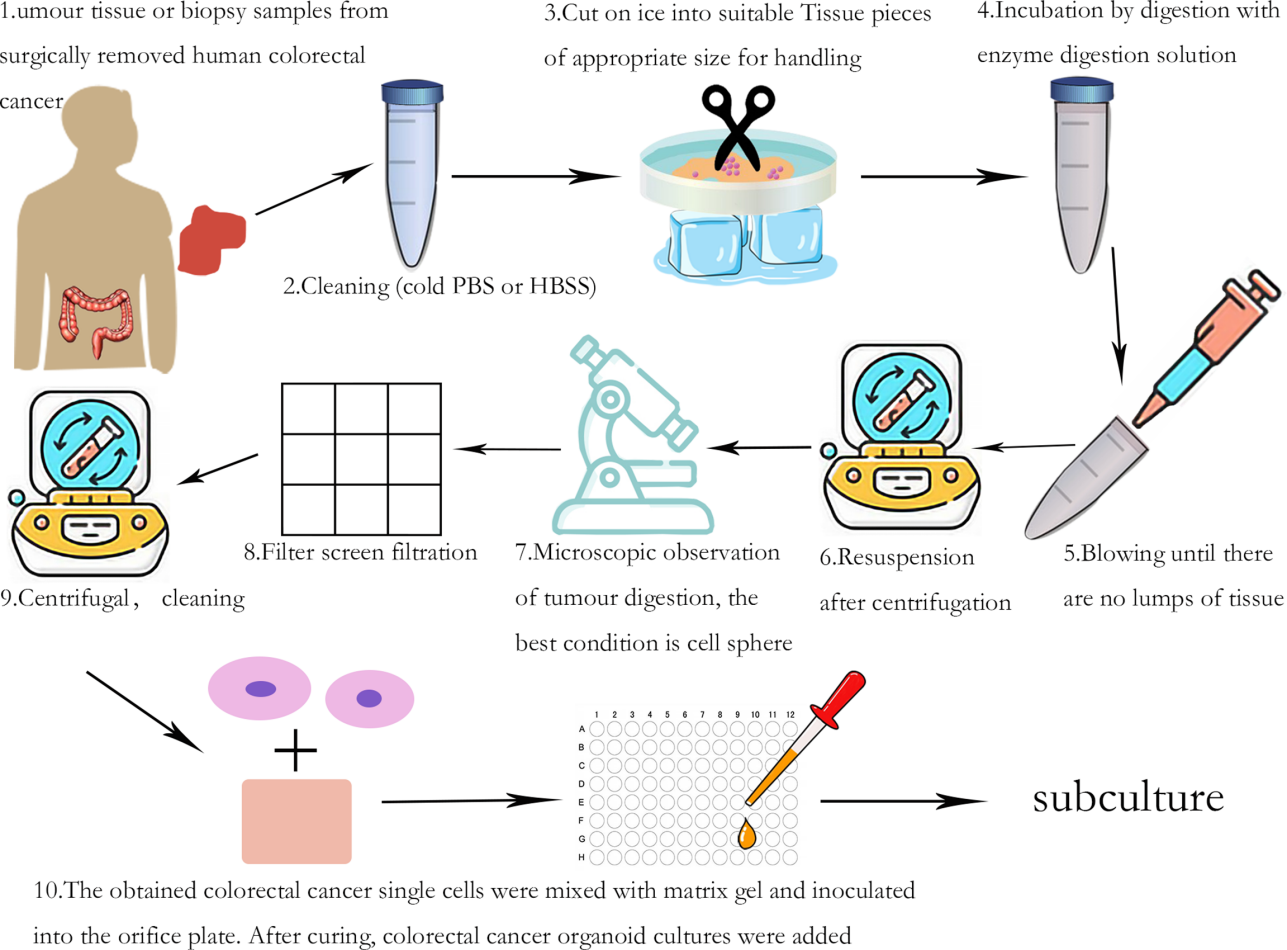
Cultivation process of PDOs.

When constructing CRC organoids, stromal environment, CRC tissue, and tissue-related digestive enzymes, basal medium (generally composed of AdDMEM/F12-HEPES-Glutamax) and growth factors that regulate signaling pathways are all necessary for culture. After preparing the required organoid materials, the culture process (**Figure 2**) needs to be strictly followed. At present, we generally use Matrigel to provide a suitable environment for single cells after operating with digestive enzymes to differentiate. Additionally, *in vitro* culture of CRC organoids requires some crucial growth factors, including epidermal growth factor (EGF), which promotes the proliferation of cancer cells. Noggin protein, a bone morphogenetic protein (BMP) inhibitor, regulates cell differentiation and prevents bone metastasis in CRC. R-spondin1, a Wnt pathway activator, promotes proliferation and metastasis of cancer cells. Prostaglandin E2 (PGE2) and nicotinamide both provide nutritional support to the organoid that maintains long-term activity. Two more compounds, A83-01 (TGF-β inhibitor) and SB202190 (p38 inhibitor), are both often used to inhibit the migration of cancer cells and help maintain the stability of the tumor organoid (12, 26). Furthermore, Y-27632 (Rho/ROCK kinase inhibitor) which can mediate cells’ functions is often used to prevent single cells from apoptosis (27, 28).

### Limitations of Construction

Up to the present day, there are still some challenges in the construction of organoids. In terms of the extracellular matrix, there are shortcomings such as high cost, complex specific composition, poor controllability, and limitations in the application. Besides, the tumor organoid after tissue implantation into Matrigel by digestive enzymes contains only epithelial cells and lacks mesenchymal cells, immune cells, and other non-cancerous cells, while the proliferation and differentiation of tumor tissues are affected by the microenvironment containing epithelium and mesenchyme (29), and the occurrence and metastasis of CRC are closely related to the epithelial–mesenchymal transition, which should be the major limitation for subsequent precise research in CRC. Moreover, the lack of mesenchymal and vascular support in the organoid makes it difficult to replicate the real intestinal organoid environment, which builds a certain obstacle to the utility of organoids in regenerative medicine, and the effect of the chemotherapy regimen of 5-fluorouracil (5-fluorouracil) in combination with Oxaliplatin, the main treatment for metastatic colorectal cancer, could not be evaluated in organoid cultured from Matrigel (30), both of which hinder the improvement of target treatments of CRC for individuals to a certain extent. In addition, it has been found that single cells dissociated from tumor tissue digestion embedded in the matrix are prone to loss of anoikis, described as programmed cell death after cell dispersion (31), which decreases the cultivating effective organoid rate due to the occurrence of apoptosis of single cells in Matrigel.

In conclusion, materials and techniques for the construction of CRC organoids still need to be optimized and constantly upgraded and created to better satisfy the practical needs of clinical implementation.

### Progress in Construction

The major milestones in culturing CRC organoids are shown in **Supplementary Figure 1**. CRC organoid studies were initiated in 2009 when Sato et al. (11) demonstrated that Lgr5 stem cells in the small intestinal crypts could be the main driver of intestinal epithelial self-renewal and that the crypt-villus structure of the small intestine could be created from a single Lgr5(+) stem cell in culture under prolonged *in vitro* culture conditions. In 2011, the team of Sato (12) optimized the prolonged culture protocols of the mouse and human colon epithelium and successfully constructed the human colon tumor organoid for the first time, opening up a new routine for the treatment of human CRC. Based on the cultural methods of the mentioned scholars, a team (32) modified to incompletely dissociate cancer tissues into tissue fragments, purify, and capture active cancer tissue cells through mesh filters and rapidly gather them to form tumor spheroid structures, then these cells were placed in Matrigel and cultured into cancer-derived organoids, which could make organoids more viable and valuable. To overcome the shortcomings of Matrigel’s lack of controllability and reliability, Gjorevski et al. (33) investigated a hydrogel, as an alternative to Matrigel, with polyethylene glycol (PEG) as the core material and Arg-Gly-Asp (RGD) adhesion sequence, and found that proliferation and differentiation states of intestinal stem cells could be achieved by modulating the softness of the hydrogel, which provides a new idea for the construction of organoids. Nicolas Broguiere et al. (34) subsequently verified that a fibronectin/laminin-based hydrogel could sustain the development of organoids for a long time, reducing the cost required for the culture substrate on the one hand and specifying the main components of the substrate that could support the growth of organoids on the other. Additionally, Usui et al. (35) firstly used collagen gel as stroma established an organoid containing epithelial and mesenchymal cells on the air–liquid interface (ALI) to better restore the intestinal epithelial response and tumor microenvironment characteristics.

With the continuous advancement of organoid technology, some scholars have also successfully established metastatic lesion organoids after CRC metastasis and verified the correlation with the primary tumor, as well as the feasibility of applying them to studies on tumor drugs and treatments. Weeber et al. (36) used metastatic biopsies from metastatic colorectal cancer to create organoids that were highly correlated with the mutated genes and DNA copy number of the primary tumor, thereby effectively reflecting the genetic characteristics of the parental tumor. The teams of Buzzelli (37) and Narasimhan (38) successfully cultured metastatic organoids from hepatic and peritoneal biopsies of CRC, respectively, and found that metastatic organoids can still ensure high success in drug screening and evaluation of treatment options after metastasis of colorectal cancer. Consequently, the successful cultivation of metastatic organoids in colorectal cancer broadens the scope of organoid research in oncology and improves the possibilities of *in vitro* models for the study of primary or metastatic cancer.

Nevertheless, incomplete replication of the models for the biological environment of the intestine as the limitation of models may not be conducive to the development of organoids in CRC; the current research (39, 40) identified an “organ-on-a-chip” model, with microfluidics as the core, which can restore the physiological environment of normal and tumor tissues in the intestine and simulate the intra-tissue vascular structures for transportation and intestinal nutrient absorption and metabolic functions. The technology creates a greater advantage for the construction of organoids and also provides an opportunity for the future application of organoids in regeneration. Furthermore, Xu et al. (41) developed a modified *in vitro* culture environment with hydroxypropyl cellulose (HPC) along with collagen as the core material, which can compensate organoids for the ability of personalized treatment assessment to some degree.

## Application of CRC Organoids

### Establishment of CRC PDO Biospecimen Bank

The prime role of a biospecimen repository is to preserve, organize, and process various types of biospecimens and maintain the biological information contained in the biospecimens to benefit clinical research. As for the different purposes of practical research, the culture of biospecimen banks can be different. The purpose of constructing a biobank on CRC organoid samples is mainly to bridge the gap between tumor genetics and patient *in vivo* experiments by verifying *in vitro* that PDOs are highly concordant with the genes and histological properties related to the source tumor and to better guide clinical use of drugs and personalized treatments by uncovering the diversity of tumor responses to drugs. Initially, van de Wetering et al. (42) have successfully established a library of 22 PDO samples, which validated the coherence of the samples with the genomic features of the primary tumors, suggesting that organoids can be used to refine the drug studies in CRC. However, the above library has some limitations for its small size and lack of organoids from rectal cancer patients and other colorectal tumors, so there is not enough objectivity in clinical studies of drug response in CRC. Fujii et al. (43) combined clinical staging and distinct types and used a medium appropriate for their source (including rectal cancer and other rare colorectal tumors) samples. A library containing 55 PDOs was successfully constructed, filling the deficiency of the former organoids and illustrating more strongly the variability of genomic characteristics and response to drugs among different tumors. Recently, Yao et al. (44) established a biospecimen bank consisting of 96 untreated locally progressive rectal cancer (LARC) PDOs, and the team of Yan HHN (45) established a biobank containing 33 early microsatellite stabilized (MSS) CRC PDOs, both of which validated organoids performed to be highly compatible with primary tumors, while differing in that the former was constructed to explore the differential effects of CRC on neoadjuvant chemotherapy, conventional radiotherapy, and the chemotherapeutic agent 5-fluorouracil and then to develop personalized patient-based regimens, while the latter was constructed to reveal novel genetic properties and new cooperation of signaling pathways driving colorectal carcinogenesis, adding a new direction to the clinical management of CRC.

In conclusion, organoids can replicate well the source tissues, and in contrast to conventional models, organoids can provide a more representative pathophysiological environment and better restore the characteristics of tumor heterogeneity. The establishment of organoid biospecimens can be more conducive to the study of tumor response to drugs at the genetic or pathway aspects, then create novel drugs for clinical therapeutic applications.

### Application in the Mechanism Research of CRC

The pathogenesis of colorectal cancer is complex and involves multiple factors, including various genetic molecules and living environments. Based on the principle that PDOs match with the cancerous tissue of origin for high-degree and relevant properties of colorectal oncogenes, it was found that after the alteration of the genomic characteristics of normal colorectal organoids, there is a tendency of dysplasia or carcinogenesis, and thus it can be inferred that the aberrant alteration of genes can induce the concurrent appearance of pathways related to CRC. The interactive pathways mainly include chromosome instability (CIN), microsatellite instability (MSI), CpG island methylator phenotype (CIMP), and sawtooth-like pathway (46). Therefore, the development of colorectal carcinoids also provides new ideas to study the mechanism of colorectal carcinogenesis and development.

The CIN pathway is the most common colorectal oncogenesis pathway by altering chromosome number and structure, thereby changing the genetic environment of the normal colorectal environment. Mutations in APC, KRAS, TP53, SMAD4, and other related genes were found to induce the CIN pathway (47). Li et al. (48) used the organoids optimized by the ALI approach and hydrogel to find malignant growth of colorectal organoids considered as the classical colorectal precancerous pathology of colorectal canal necrosis and serrated epithelial lesions after using Cre recombinase to edit APC, KRAS, TP53, and SMAD4. After long-term passaged culture, CRC started to appear. Meanwhile, this team also found that APC mutation, together with miR-483 overexpression in normal organoids, could also induce CRC growth. Thus, we can speculate that the culture of CRC organoids can reflect the association between oncogenesis and multiple genes and can confirm the close correlation between CIN and colorectal carcinogenesis. In addition, the teams of Drost (49) and Roper (50) used another gene-editing technique, CRISPR-Cas9, to mutate the oncogenes APC and TP53 in the organoid tissue, and elucidated that the invasive CRC arose from the position where edited organoid transplanted into mice. Bolhaqueiro *et al*. (51) observed directly by culturing PDOs that the chromosome number and structure of the daughter cells divided from tumor cells were not normal, which can verify that the CIN pathway can mediate colorectal carcinogenesis and also validate the important role of colorectal organoids in probing the mechanisms of colorectal carcinogenesis.

Microsatellite instability (MSI), especially high (MSI-H), occurs inextricably with DNA mismatch repair (MMR) gene silencing, commonly MLH1, MSH2, MSH6, and PMS2 (52). In contrast, colorectal carcinogenesis is often strongly associated with the MSI-H pathway. Mechanisms, like the aberrant induction of the MAPK/ERK signaling pathway caused by BRAF ^V600E^ mutation (53) and abnormal TGF-β signaling pathway caused by transforming growth factor-β receptor II (TGF-βRII) mutation (54), often trigger the MSI pathway. Reischmann N team (55) verified that BRAF ^V600E^ mutations could lead to tissue disintegration and cell death in colonic organoids, thus indicating that BRAF ^V600E^ mutation CRC has a poor prognosis. In their exploration, what we need to pay more attention to is that BRAF ^V600E^ mutant organoids, together with the CIN pathway-associated gene mutations APC deletion and TP53 mutation in coculture, could reduce ERK/MAPK pathway activity, which improved the organoid environment and restored the growth and proliferation function of cells in BRAF ^V600E^ mutant organoids to some extent. It suggests more possibilities for the interactions among mutant genes associated with both MSI and CIN to influence CRC progression and provides new directions for CRC drug therapy. The team of Takeda (56)established that organoids are containing TGF-βRII mutations incorporating CRISP-Cas9 and found that TGF-βRII mutations could act in conjunction with activin receptor (ACVR) mutations in the TGF-β pathway, resulting in abnormalities in the TGF-β signaling pathway, and verifying that TGF-βR II synergizes with ACVR mutations in colorectal cancer. It has been shown (57) that 90% of MSI CRC are attributed to abnormal TGF-β signaling pathway, so it can be speculated that TGF-βR II and ACVR mutations in the TGF-β pathway have a synergistic effect on MSI CRC, which further clarifies the mechanism of MSI colorectal carcinogenesis and adds more solution for challenges of the MSI CRC clinical therapeutic selection of drugs.

As unique colorectal carcinogenesis pathways, both CIMP and the serrated pathway are relevant in most cases. CIMP, generally interpreted as CpG island methylation, inhibits the normal transcription of genes and thus downstream genes for their normal expression. It is mostly positively expressed in serrated colorectal adenomas, often occurring in collaboration with both BRAF mutations and MSI (58). The Lannagan team (59) applied CRISPR-Cas9 editing technology to construct BRAF^V600E^ as well as MLH1 mutation organoids and successfully modeled the characteristic lesions related to serrated colorectal adenocarcinoma, such as infiltrative growth of serrated tissue with mucus cap, and detected an increased frequency of CpG methylation, which verified that the serrated pathway activation has a strong correlation with BRAF ^V600E^, MSI, and CIMP. It can be supposed that the organoid can still maintain high fidelity to the CRC environment, which provides a piece of important evidence for extensively studying the molecular pathways of CRC in the future.

In summary, CRC organoids are competent to recapitulate the relationship between genes and signaling pathways and validate the complexity, diversity, and association between pathway mechanisms of oncogenesis. Applications of tumor organoids can promote research in predicting the development of CRC prognosis based on genetics and in innovating oncology drugs to regulate the interaction among pathways.

### Predicting Drug Response and Screening for Antitumor Drugs

Due to the phenomenon of tumor heterogeneity, the response of tumors induced by different pathways to drugs may also differ, so the development of various antitumor drugs is inseparable from the study of tumorigenesis and development mechanisms. Furthermore, the preclinical, experimental stage is necessary to ensure that the efficacy of drugs in clinical applications can still achieve the expectation in basic research, which requires the support of a majority of suitable models. On the one hand, CRC PDOs can be used to establish a biological sample bank and preserve the heterogeneity of CRC, providing abundant preclinical models that can be used to predict drug response and efficacy; on the other hand, organoids can be functional in estimating the effect of drugs on genes or signaling pathways referring to the mutation characteristics of colorectal oncogenes and screen out suitable anti-CRC drugs referring to patients’ clinical–pathological data.

Schütte et al. (60) collected tumors from 106 colorectal cancer patients, constructed a biospecimen library containing 35 PDOs and 59 PDXs, and evaluated and compared the response between them to drugs by IC50/Emax. The two models were in agreement to be severely resistant to the chemotherapeutic agent oxaliplatin and be relatively sensitive to the EGFR inhibitor afatinib, but there was a significant disparity in the drug response to both 5-fluorouracil and AZD8931, suggesting that the environment of the preclinical model correlated with the drug response. Additionally, by analyzing the difference in sensitivity to the drug between PDOs with different mutations in the RAS/RAF pathway, it is suggested that the different mechanisms of colorectal carcinogenesis affect the effect of drug treatment, and it is also possible to confirm the high reduction of organoids compared to tumors. Verissimo et al. (61) constructed KRAS wild-type versus KRAS mutant CRC organoids edited by CRISPR-Cas9 for assessment of the EGFR-RAS-ERK pathway drugs and the EGFR inhibitor afatinib alone or in combination with the MEK inhibitor selumetinib and found that the combination strengthened the effect on KRAS wild-type tumors that were sensitive to only afatinib previously, but still there was no significant efficacy in the KRAS mutant-type, which was caused by the fact that this combination of inhibitors only stalled the cell cycle but did not kill cells. Organoids are informative to apply for drug treatment studies in the abnormal RAS pathway in CRC. The organoid also plays an important role in CRC drug research regarding the WNT/β-catenin pathway. Some scholars (62) found that aberrant activation of WNT/β-catenin was associated with TP53 mutations after treatment with 5-fluorouracil through CRC PDOs, explaining the possibility that most colorectal cancers are resistant to treatment with 5-fluorouracil alone. However, by further exploration, it was found that Wnt inhibitors with 5-fluorouracil can reduce tumor cell re-aggregation and reduce the probability of CRC recurrence. Spit *et al*. (63) showed that tumorigenic RNF43 mutations exhibited synergistic effects with TP53 mutations and were more resistant to treatment with Wnt inhibitors, which differed from RNF43 loss-of-function mutations, through the culture of CRC organoids.

In conclusion, organoids have great potential for drug sensitivity testing. When combined with their analysis of colorectal cancer gene expression, organoids can help investigate targeted drugs at the genetic level and evaluate the amount of new treatment options.

### Personalized Treatment as the New Direction

Personalized medical decisions for therapy are made in pursuit of optimal therapeutic outcomes, based on the patient’s genetic and proteomic profiles, combined with the individual metabolic environment, to target patient-specific regimens. Drugs that target genes in precision medicine often inhibit tumor growth and prevent tumor metastasis by effectively interfering with signaling pathways (64). The high content of consistency between organoids and the original tumors, in terms of morphology, genotype, specific functions, mutational features, and physiological and pathological transformations, provides great support for the establishment of personalized medicine systems.

Pauli et al. (65) established KRAS and TP53-mutated stage IV CRC organoids and APC-mutated stage IV CRC organoids and found significant differences in sensitivity to different drugs between those mutated-type organoids, with the former responding only to the MEK inhibitor trametinib, while the latter was sensitive to the afatinib–histone deacetylase inhibitor combination and was more sensitive than the conventional colorectal cancer treatment regimen FOLFOX (oxaliplatin, 5-fluorouracil, and leucovorin), thus suggesting that a patient-specific dosing regimen based on patient pathologic records would be more beneficial to against the tumor. Tashiro *et al*. (66) through constructs of KRAS wild-type and mutant-type organoids *in vitro* found that patients with different KRAS mutations differed significantly from each other in response to cetuximab. Continuously, those models combined with trametinib were used to indicate that trametinib could enhance the drug response in patients who could benefit from cetuximab, resulting in more significantly beneficial responses to those patients but no significant therapeutic improvement in the tissues of CRC patients who had performed resistance to cetuximab. The above studies reflect that patient tumor heterogeneity increases the possibility of drug selection, thus allowing treatment options to be diverse and individualized.

In recent years, CRC immunotherapy has developed rapidly, and research on immunotherapy targets has made great progress. By showing the differences between the genomic traits of each patient and the variety of regulatory effects of different immune pathways on the tumor microenvironment, tumor organoids can then seek breakthroughs from gene combination immunotherapy targets for patients and develop personalized treatment plans for patients. Currently, programmed cell death protein-1 (PD-1) and programmed cell death-ligand 1 (PD-L1) are popular immunotherapeutic targets in CRC (67). By incubating PD-L1-overexpressing PDOs with the PD-1 inhibitor nivolumab, they found that nivolumab induced increased CD8+ T-cell infiltration, decreased PD-L1 expression, and reduced tumor cell proliferation, but its therapeutic effects were concentration-dependent, leading to the hypothesis that nivolumab could be used as a PD-1-targeting agent. Combined with the PD-L1 expression in each PD-L1-positive patient, an immunotherapy regimen would develop appropriately for patients. As an LRP5/LRP6 receptor-binding protein that inhibits Wnt/β-catenin pathway activity, Dickkopf (DKK) has a therapeutic effect on CRC with aberrant activation of the Wnt/β-catenin pathway (68), and the team of Sui (69), in an MSI/dMMR CRC PDO-based study, observed that DKK1 inhibited the activity of PD-1 inhibitors, mainly by limiting the immune function of CD8+ T cells and increasing tumor cell activation. It suggested that blockade against DKK1 protein could be an option of elevating the impacts of PD-1 inhibitors in immunotherapy. We may realize that personalized therapeutic regimens combining regulatory factors on signaling pathways with immunotherapeutic targets, to some extent, attenuate the possible risk of drug resistance or immune limitation in treatment, in which the highly reductive effect of organoid on tumor microenvironment plays an essential role in the study.

Up to now, as the concept of personalized medicine is gradually kept in mind, the space for organoid technology continues to grow. The use of CRC organoid culture technology has broadened the horizons of diagnosis and treatment of CRC and reminds us to target more breakthroughs in medical healthcare and drug selection of CRC.

## Conclusion

Major progressions of organoids from 2009 to 2020 are supplemented in **Supplementary Figure 1**. Although there are still many limitations in the research of organoids, such as being costly, time-consuming, and labor-intensive; requiring sufficient patient-derived tumor and tissue samples; and having less mature technology, several of them have been solved or improved with the development and advances of medicine and technology (32–35, 39–41). To utilize the functions of organoids better and accelerate their application in clinical therapy, more and more investigations about tumor organoids would provide promising rationales and directions in the war against cancer in the future.

## Author Contributions

JL, XH, BZ, and WT conceived of the study and participated in its design and coordination and helped to draft the manuscript. JL, XH, LH, JH, DL, LL, YD, LZ, BZ, and WT made a significant contribution to the work about the analysis, writing, and interpretation. All authors contributed to the article and approved the submitted version.

## Funding

This research was funded by the Guangxi Clinical Research Center for Colorectal Cancer (Guike: AD19245197); Research Basic Ability Improvement Project for Guangxi Young College Teachers (2021KY0087); Innovation Project of Guangxi Graduate Education (YCSW2021133); Guangxi Medical University Autonomous Region-level College Student Innovation Training Project (202110598177); Guangxi Natural Fund Facial Project (2021JJA140081); Guangxi Medical and Health Appropriate Technology Development and Promotion Project (S2021016); and The project of improving the basic ability of scientific research of young and middle-aged teachers sponsored by the Education Department of Guangxi Zhuang Autonomous Region (2021KY0086).

## Conflict of Interest

The authors declare that the research was conducted in the absence of any commercial or financial relationships that could be construed as a potential conflict of interest.

## Publisher’s Note

All claims expressed in this article are solely those of the authors and do not necessarily represent those of their affiliated organizations, or those of the publisher, the editors and the reviewers. Any product that may be evaluated in this article, or claim that may be made by its manufacturer, is not guaranteed or endorsed by the publisher.

## Supplementary Material

Supplementary Figure 1 Major progressions of CRC organoids. The red represent major milestones in culturing CRC organoids; the green represent major findings based on CRC organoids.

## Abbreviations

CRC, colorectal cancer; CSC, colorectal stem cell; PDX, patient-derived xenograft; PDOs, patient-derived organoid; OPC, oligomeric proanthocyanidin; PEG, polyethylene glycol; RGD, Arg-Gly-Asp; EOCRC, early-onset colorectal cancer; LARC, locally progressive rectal cancer; CIN, chromosome instability; DKK1, Dickkopf-related protein-1; MSI, microsatellite instability; CIMP, CpG island methylator phenotype.
